# Productive chaos and precision engineering: decoupling discovery from manufacturing to revolutionize plant-inspired therapeutics

**DOI:** 10.3389/fpls.2026.1771802

**Published:** 2026-03-06

**Authors:** Dexter Achu Mosoh

**Affiliations:** 1 Department of Biomedical Engineering, Indian Institute of Technology Ropar, Rupnagar, Punjab, India; 2 Professor Wagner A. Vendrame’s Laboratory, Horticultural Sciences Department, University of Florida, Institute of Food and Agricultural Sciences, Gainesville, FL, United States

**Keywords:** artificial intelligence, biofoundries, cofactor balancing, hairy root culture, metabolic engineering, microbial cell factories, P450 engineering, plant natural products

## Abstract

The pharmaceutical industry remains critically dependent on plant-derived natural products, yet the supply of these complex molecules is perpetually threatened by the inherent biological instability of plant systems. For decades, the field has struggled to force undifferentiated plant cell cultures into the mold of consistent industrial fermentation, a strategy largely defeated by intrinsic biological stochasticity arising from epigenetic reprogramming, somaclonal variation, transcriptional noise, and systemic metabolic rigidity, as well as by a linear cost structure that prohibits pharmaceutical scalability. This literature-based review articulates a fundamental paradigm shift: the strategic decoupling of discovery from production. It argues that the genomic and epigenomic plasticity of plant cells—rather than being suppressed—should be deliberately induced and explored through stress elicitation to generate a “productive chaos” of chemical diversity for discovery. This expanded metabolic landscape is then decoded using single-cell–resolved multi-omics and spatial metabolomics to identify rare, elite producer states, alongside advanced artificial intelligence, molecular networking, and structure prediction to characterize novel bioactive candidates. Once identified, these biosynthetic pathways are functionally repatriated into defined, heterologous microbial hosts, engineered via systems-level metabolic and architectural optimization—including cofactor balancing, dynamic pathway control, subcellular compartmentalization, and cytochrome P450–reductase stoichiometry—to achieve stable, high-titer manufacturing. By integrating high-throughput discovery, AI-guided strain design, techno-economic analysis, and regulatory Quality-by-Design principles, this discovery–production decoupling resolves the long-standing tension between biological complexity and industrial rigor. This framework transforms the economics of natural product supply, transitioning from the low-CAPEX/high-OPEX trap of extraction to the high-CAPEX/low-OPEX scalability of fermentation, offering a scientifically grounded, commercially viable, and regulatorily tractable pathway to unlock the full therapeutic potential of the plant kingdom.

## Introduction

The plant kingdom remains the most prolific enzymatic engineer on the planet, serving as the origin for a significant proportion of small-molecule therapeutics approved over the last four decades (Newman & Cragg, 2020). From the complex diterpenoid paclitaxel to the sesquiterpene artemisinin, plant natural products (PNPs) provide structural complexity and bioactivity that synthetic chemistry continues to struggle to replicate (Cragg, 1998; Mosoh, 2024b, 2024a, 2025; Mosoh & Vendrame, 2025; Paddon et al., 2013; Yin et al., 2025). However, the pharmaceutical utility of these molecules is perpetually constrained by the so-called “supply problem”: the reliance on slow-growing, environmentally sensitive agricultural sources or extraction from wild populations, practices that are often economically, ecologically, and geopolitically unsustainable (Mosoh et al., 2024d). Economically, this reliance entrenches a low-CAPEX/high-OPEX production model, in which costs scale linearly with biomass and downstream purification, rendering many high-purity therapeutics prohibitively expensive (R. Lynd et al., 2022).

For nearly half a century, the biotechnology sector has attempted to solve this supply crisis by force-fitting undifferentiated plant cells into industrial fermentation paradigms, aiming to replicate the scalability and predictability of microbial systems. Despite substantial investment, this approach has largely failed to deliver a generalized platform for PNP manufacturing. As increasingly revealed by epigenomic, transcriptomic, and metabolic analyses, plant cell cultures are intrinsically unstable systems, characterized not only by epigenetic drift and somaclonal variation, but also by stochastic gene expression, dynamic chromatin architecture, and tightly coupled metabolic networks that actively resist deterministic control (Davies, 2010; Mosoh et al., 2024c). Consequently, the field faces a fundamental dichotomy: the chemical ingenuity of the plant is indispensable for discovery, yet the plant cell itself is often an unsuitable chassis for industrial production (Ke and Yoshikuni, 2020; Van Dien, 2013).

We are now at a technological inflection point. The convergence of single-cell omics, high-resolution and spatial metabolomics, artificial intelligence (AI), and synthetic biology offers a route to resolve this long-standing tension. Rather than treating biological variability as a defect to be minimized, emerging strategies deliberately harness stress-induced metabolic heterogeneity as a discovery engine, provoking cryptic biosynthetic pathways and expanding accessible chemical space. This “productive chaos” can now be systematically decoded using AI-driven molecular networking, structure prediction, and single-cell–resolved analyses that isolate rare, high-performing cellular states (Beniddir et al., 2021; Mullowney et al., 2023). 

Once a bioactive candidate and its biosynthetic logic are identified, the manufacturing challenge is decisively decoupled from the discovery source. Production is transferred to defined, heterologous microbial hosts—such as *Saccharomyces cerevisiae*, *Yarrowia lipolytica*, or *Streptomyces* spp.—that can be rationally engineered using predictive computational models, dynamic pathway control, and subcellular architectural optimization (Choi et al., 2019; Zhang M. et al., 2023; Zhang T.-L. et al., 2023). While differentiated plant tissues, such as hairy roots, may serve as a pragmatic alternative for particularly recalcitrant pathways, the future of scalable pharmaceutical manufacturing lies in precision-designed microbial platforms insulated from plant-derived biological instability.

This literature-based review articulates a paradigm shift in plant biotechnology: the strategic decoupling of discovery from production. The limitations of the historical “control paradigm” in plant cell culture are first dissected at genetic, epigenetic, transcriptional, and metabolic scales (Sections A–B). Stress elicitation is then reframed as a systematic engine for chemical discovery (Section C), enabled by an AI-powered analytical toolkit capable of navigating high-dimensional metabolomic data (Section D). Finally, pragmatic blueprints are presented for repatriating validated pathways into stable production hosts (Section E), and for closing the design–build–test–learn loop using predictive computational design (Section F), culminating in a regulatory-ready, economically sustainable high-CAPEX/low-OPEX manufacturing model (Section G).

By transitioning from a reactive struggle against biological complexity to a proactive, data-driven design philosophy, this framework redefines how plant-derived therapeutics can be discovered, manufactured, and commercialized in the modern era.

## Section A: the illusion of control—intrinsic biological instability in plant systems

The historical ambition of plant cell fermentation has been predicated on the concept of the “factory in a flask”—a sterile, homogeneous bioproduction system capable of delivering consistent yields of specialized metabolites. This paradigm assumes that biological variation is a technical failure resulting from suboptimal process control. However, despite decades of optimization in media composition and bioreactor design, instability in growth, productivity, and genetic fidelity remains a persistent bottleneck in commercial micropropagation and secondary metabolite production (Majumder et al., 2025; Mosoh et al., 2023, 2024a, b; Mosoh et al., 2024). Emerging evidence from systems biology, single-cell transcriptomics, and metabolic flux analysis suggests that this instability is not merely an engineering deficiency but a fundamental property of plant biological systems (Keurentjes et al., 2011; Maranas and Nemhauser, 2025). Furthermore, the organization of specialized metabolic pathways into biosynthetic gene clusters (BGCs)—genomic hotspots of rapid evolution and regulation—introduces a layer of structural and regulatory complexity that actively resists enforced uniformity (Bharadwaj et al., 2021; Cawood and Ton, 2025). The interactions between epigenetic drift, stochastic gene expression, and metabolic network rigidity render the quest for absolute determinism in *in vitro* systems a strategic misstep.

### The myth of the clonal line: somaclonal variation

The foundational assumption of the control paradigm is the stability of the clonal line—the idea that asexual propagation maintains genetic and phenotypic uniformity. However, the process of dedifferentiation required for callus induction acts as a catalyst for significant genomic and epigenomic divergence, a phenomenon termed somaclonal variation (Mosoh et al., 2024a; Mosoh and Vendrame, 2025; Pawełkowicz et al., 2021).

Recent whole-genome bisulfite sequencing (WGBS) has revealed that the transition from differentiated leaf tissue to pluripotent callus involves massive epigenetic reprogramming. For instance, in *Prunus persica* (peach), this transition is accompanied by global DNA hypomethylation and the removal of the repressive histone mark H3K27me3, specifically at promoters of auxin- and cytokinin-related regulators (Zheng B. et al., 2022). Similarly, in *Hordeum vulgare* (barley), callus induction triggers distinct methylation changes in CG, CHG, and CHH contexts, which directly correlate with the transcriptional activation of regeneration-associated genes such as *HvWOX5* and *HvLBD16* (Huang et al., 2025).

This epigenetic fluidity is not benign; it introduces significant heterogeneity into the culture. In *Arabidopsis thaliana*, the leaf-to-callus transition is marked by a specific reduction in CHH methylation within transposable element (TE) regions (Shim et al., 2022). This hypomethylation compromises genome integrity, potentially allowing TE activation and relocation, which serves as a primary driver of somaclonal variation (Shim et al., 2022). Crucially, BGCs—which encode high-value specialized metabolites—are frequently enriched with TEs and reside in dynamic chromosomal regions, such as subtelomeric zones, that are prone to recombination and rearrangement (Cawood and Ton, 2025; Smit and Lichman, 2022). Under the stress of *in vitro* culture, the desilencing of these TEs can catalyze genomic restructuring within these clusters, accelerating chemical diversification but undermining clonal stability (Cawood and Ton, 2025). Indeed, these “dynamic genomic neighborhoods” act as evolutionary playgrounds where gene duplication and neofunctionalization occur rapidly, challenging the preservation of a static genotype required for industrial standardization (Smit and Lichman, 2022). Furthermore, transcriptome profiling of *Cucumis sativus* somaclones has demonstrated that these molecular changes are random and distributed across all chromosomes (Pawełkowicz et al., 2021). Therefore, a genetically “identical” starting material is merely a transient snapshot; the innate drive for divergence during dedifferentiation renders the stable “clonal line” an unstable concept (dos Santos Ferreira et al., 2023).

### The failure of deterministic control: stochastic gene expression

Even if genomic and epigenomic drift could be arrested, the control paradigm faces a deeper, intrinsic hurdle: transcriptional noise. Contrary to the model of continuous, analog gene expression, transcription in living cells occurs in discontinuous “bursts” or pulses (Chubb and Liverpool, 2010; Rodriguez and Larson, 2020). This phenomenon, termed transcriptional bursting, results in significant variability in mRNA abundance between genetically identical cells sharing the same environment (Tunnacliffe and Chubb, 2020).

This variability is exacerbated by the complex regulatory architecture of BGCs. BGC expression is tightly coordinated by specific chromatin signatures, including the enrichment of the repressive mark H3K27me3 and the histone variant H2A.Z, which antagonistically regulate access to DNA (Bharadwaj et al., 2021; Cawood and Ton, 2025). The physical clustering of these genes facilitates their regulation within Topologically Associated Domains (TADs), where long non-coding RNAs (lncRNAs) and super-enhancers modulate chromatin loops to synchronize expression (Cawood and Ton, 2025; Méteignier et al., 2023). While this allows for potent stress responses, it implies that small fluctuations in chromatin state can switch entire metabolic pathways on or off stochastically across a cell population, creating heterogeneity that is difficult to control in a bioreactor.

Recent single-molecule imaging and RNA sequencing technologies have quantified this variability in plant systems. In *Arabidopsis*, gene expression noise is pervasive and can be functional, allowing plants to employ bet-hedging strategies against environmental fluctuations (Cortijo and Locke, 2020). However, within the context of a bioproduction reactor, this noise manifests as heterogeneity in yield. The mechanism of this variability is linked to chromatin architecture. Gene body methylation (gbM) has been identified as a buffer for transcriptional noise; genes lacking gbM display significantly higher expression variability (Zastąpiło et al., 2024). Furthermore, dynamic DNA methylation turnover in gene bodies is associated with enhanced expression plasticity, suggesting that the epigenetic state directly modulates the “dynamic range” of transcription (Williams et al., 2023). Consequently, providing a uniform bioreactor environment is insufficient to ensure uniform productivity. Molecular processes are inherently probabilistic; features such as upstream open reading frames (uORFs) have evolved specifically to mitigate this noise and ensure precise protein levels for critical regulators like *TOC1* (TIMING OF CAB EXPRESSION 1) (Wu et al., 2022). Without such buffering, cell-to-cell heterogeneity is inevitable, leading to populations where only a fraction of cells may be actively producing the target metabolite at any given time (Maranas and Nemhauser, 2025; Usaj et al., 2021).

### The futility of isolated engineering: systemic metabolic complexity

Finally, even if gene expression were perfectly synchronized, the metabolic network itself resists simplified manipulation due to its inherent rigidity and interconnectivity. Metabolic flux analysis (MFA) reveals that plant secondary metabolism is not a linear assembly line but a complex grid of competing pathways and subcellular compartments (Shih and Morgan, 2020).

The concept of the “metabolic sink” illustrates the homeostatic resistance of these networks. For example, the introduction of tryptophan decarboxylase (TDC) into potato tubers created a sink for tryptophan that drastically altered the phenylpropanoid pathway, reducing the levels of chlorogenic acid and compromising resistance to *Phytophthora infestans* (Yao et al., 1995). Similarly, ectopic expression of jasmonic acid O-methyltransferase (*AtJMT*) in *Nicotiana attenuata* created a metabolic sink that depleted internal jasmonic acid (JA) and JA-isoleucine pools, effectively silencing downstream defense genes like threonine deaminase (Stitz et al., 2011).

This resistance is further complicated by the physical organization of pathways into BGCs, which function to prevent the accumulation of toxic intermediates through tight genetic linkage and co-inheritance (Smit and Lichman, 2022). For instance, the fragmentation of the avenacin cluster in oat or the α-tomatine cluster in tomato leads to the build-up of cytotoxic precursors, triggering negative selection pressures that preserve cluster integrity (Cawood and Ton, 2025). Furthermore, metabolic channeling and enzyme assemblies (metabolons) sequester intermediates and prevent them from equilibrating with the bulk phase (Pareek et al., 2021; Sweetlove and Fernie, 2018). Recent computational tools like plantiSMASH and PhytoClust have revealed that such complex clustering is widespread, encoding diverse classes of metabolites including terpenoids, alkaloids, and benzoxazinoids (Kautsar et al., 2017; Töpfer et al., 2017). Consequently, engineering a single pathway often fails because the metabolic network’s inherent rigidity, enforced by genomic clustering and toxic intermediate avoidance strategies, compensates, diverts, or resists the targeted manipulation (Li and Van Eck, 2007; Smit and Lichman, 2022).

Therefore, the evidence from somaclonal, transcriptional, and metabolic scales converge on a single conclusion: variability and instability are not exceptions to be engineered away but fundamental properties of plant biological systems. The “Illusion of Control” is the failure to recognize this reality. The interactions between epigenetic reprogramming during callus formation, the stochastic nature of transcriptional bursting regulated by dynamic chromatin domains, and the compensatory mechanisms of metabolic networks anchored in biosynthetic gene clusters ensure that heterogeneity is the rule, not the exception. The old paradigm of fighting this instability is a strategic misstep; modern technologies now allow us to characterize this “productive chaos” to harness it rather than suppress it.

## Section B: the sisyphean endeavor—when the control paradigm meets economic reality

Having established that intrinsic biological instability is a fundamental property of plant systems (Section A), we now examine its consequences in the real world. The attempt to enforce control through bioprocess engineering does not eliminate this instability; instead, it amplifies it, creating a “Sisyphean Endeavor” where gains in productivity are continually eroded by the compounding forces of scale-up physics and downstream economics.

### The scale-up mirage: amplifying biological instability

The first barrier emerges during scale-up. The well-mixed, homogeneous environment of a laboratory shake flask is a fantasy in a large-scale bioreactor. As vessel volume increases, maintaining mass transfer coefficients (k_L_a) comparable to bench-scale systems becomes increasingly energy-intensive and physically damaging to the cells (Chattopadhyay et al., 2002). Plant cells are distinct from microbial hosts; they are large (20–100 µm), vacuolated, and notoriously sensitive to shear stress (Namdev and Dunlop, 1995).

In an industrial bioreactor, the hydrodynamic forces required for adequate mixing and oxygen transfer create a heterogeneous environment of shear zones and stagnant eddies (Joshi et al., 1996). These physical stresses do not merely affect growth; they interact with the genetically and epigenetically dynamic cell population described in Section A. Hydrodynamic stress acts as an unpredictable selective pressure, favoring cell lines with robust cell walls or aggregated phenotypes over those with high metabolic flux (Chattopadhyay et al., 2002). Furthermore, “shear-induced elicitation”—where mechanical stress triggers defense pathways unpredictably—can alter the metabolic profile mid-batch, leading to batch-to-batch inconsistency that defies regulatory standards (Namdev and Dunlop, 1995). Consequently, the bioreactor does not just house the biological system; it actively and unpredictably selects against the “stable” clonal line the process was designed to maintain.

### The economic bottleneck: the unsustainable cost of purity

Even if a moderately stable upstream process is achieved, a more formidable barrier awaits: cost. The core objective of the control paradigm—to produce a single, pure compound from a crude cell lysate—runs headlong into the reality of downstream processing (DSP). Techno-economic analyses (TEA) consistently reveal that for intracellular metabolites produced at low titers (<1 g/L), DSP can constitute 50–80% of the total Cost of Goods Sold (COGS) (Lynch, 2021; Salas-Villalobos et al., 2024). This cost is driven by the sheer complexity of the plant matrix, which requires extensive solvent usage and multi-step purification to remove abundant co-metabolites and cellular debris (Nandi et al., 2016). Consequently, without the high titers characteristic of microbial fermentation, plant cell culture remains trapped in a cost structure where purification expenses overwhelm the value of the product (Lynd et al., 2022).

This creates a fatal negative feedback loop. Biological instability and strict metabolic regulation keep volumetric productivity low (typically mg/L rather than g/L). Low titers mean that vast volumes of biomass and media must be processed to recover small amounts of product (Verpoorte et al., 2002). This exponentially increases the burden on extraction and purification unit operations (e.g., chromatography, crystallization), which are capital-intensive and suffer from poor economies of scale (Pizarro Carbajal, 2022). For instance, modeling of resveratrol production demonstrates that while bioreactor costs are significantly high, the inability to achieve high titers shifts the economic center of gravity entirely to purification, driving the minimum selling price (MSP) to levels (> $150 USD/kg) that are non-competitive with chemical synthesis or extraction from field-grown biomass (Pizarro Carbajal, 2022). In contrast, microbial fermentation platforms, such as those utilizing *Trichoderma reesei* for enzyme production, have demonstrated that optimized bioprocesses can achieve enzyme costs as low as $3.2 USD/kg, significantly outperforming traditional plant-based systems when high titers are achievable (de Lima et al., 2022). The pursuit of purity from a dilute, complex matrix becomes economically suicidal for all but the highest-value molecules.

### Case studies in exceptionalism: the rule-proving exceptions

Skeptics of this pessimistic view often point to the celebrated commercial successes of shikonin and paclitaxel (Taxol^®^) as evidence that the control paradigm is viable. However, a critical dissection reveals these to be exceptions that prove the rule (Cragg, 1998).

Shikonin, the first commercial product from plant cell culture, succeeded because it is a red pigment secreted into the medium (simplifying DSP), has a high market value as a dye and pharmaceutical, and its production could be decoupled from growth in a two-stage process (Malik et al., 2016; Yazaki, 2017). Similarly, paclitaxel’s success relied on a perfect storm of non-replicable conditions: an exceptionally high-value target (a blockbuster anticancer drug), a specific elicitation strategy (methyl jasmonate) that reliably boosts yields, and massive, sustained investment from Bristol-Myers Squibb to optimize the cell line over decades (Cragg, 1998; Yin et al., 2025). Recent genomic elucidations have further enabled the identification of a minimal gene set for paclitaxel biosynthesis, allowing for potential heterologous production in microbial hosts or *Nicotiana benthamiana*, although yields remain below commercial viability without extensive optimization (Coombe-Tennant et al., 2025).

Contrast these with the “silent majority” of failures. Attempts to commercialize other high-value compounds like vincristine, vinblastine, or complex terpenoids via cell culture have largely languished in the “valley of death” (Isah et al., 2018). In these cases, biological instability could not be overcome by simple elicitation, and the resulting low yields made the downstream economics untenable (Guo et al., 2025; Wu et al., 2021). Even in successful cases like paclitaxel, long-term cell cultures suffer from epigenetic silencing of key biosynthetic genes, necessitating continuous and costly re-selection or treatment with demethylating agents to maintain productivity (Coombe-Tennant et al., 2025). The historical record demonstrates that the control paradigm is not a reliable platform technology but a high-stakes gamble suitable only for a minute fraction of plant metabolites.

Thus, the scale-up challenges amplify biological instability, the downstream economics make low-yield processes untenable, and the historical record shows that success is a prohibitively expensive anomaly. The combined weight of this evidence leaves no doubt: the traditional control paradigm, based on fighting biological instability to produce a single compound, is a strategic misstep that leads to commercial non-viability (**Box 1**).

Box 1 **The mechanism of instability: Epigenetic drift and somaclonal variation in plant cell culture**

While plant cell fermentation was initially envisioned as a scalable alternative to agricultural harvesting, it is fundamentally limited by somaclonal variation—a phenomenon where cultured cells accumulate genetic and epigenetic changes over time, leading to the loss of biosynthetic capacity (Mosoh and Vendrame, 2025). Unlike microbial hosts, which can be maintained as stable master cell banks, undifferentiated plant cells (callus or suspension) are prone to rapid phenotypic drift due to the high stress of the *in vitro* environment.

Epigenetic Silencing: A primary driver of yield instability is the hypermethylation of biosynthetic gene promoters. For example, in *Taxus* species (the source of paclitaxel), long-term culture often results in the progressive transcriptional silencing of key pathway enzymes, such as taxadiene synthase, despite the structural integrity of the genes remaining intact (Zhang Y. et al., 2023). This epigenetic drift is exacerbated by the dedifferentiation process, which strips the genome of the regulatory architecture found in organized tissues (Wu et al., 2021).

Chromosomal Instability: Plant cells in suspension often exhibit ploidy instability and chromosomal rearrangements. Without the selection pressure of maintaining whole-plant organismal integrity, cells that divert energy away from costly secondary metabolism toward primary growth gain a fitness advantage. Over successive sub-cultures, these non-producing “cheater” cells outcompete high-producers, leading to the collapse of the production line (Moses et al., 2013; Wilson and Roberts, 2012).

Technological Implication: This instability renders the “force-fitting” of plant cells into industrial fermenters a Sisyphean task. Rather than fighting this inherent plasticity, the new paradigm leverages it solely for discovery (where transient diversity is an asset) while delegating stable production to engineered heterologous hosts (Section E).

Therefore, we are compelled to seek a new strategy. The futility of the old way is clear. But what if, instead of fighting complexity, we could harness it? This new approach is no longer a fantasy, because the same technologies that revealed the depth of the problem—omics, real-time analytics, data science—now provide the tools to build a solution.

## Section C: the paradigm shift—harnessing variability as a discovery engine

Sections A and B established a stark reality: the intrinsic instability of plant systems makes the traditional “control paradigm” a biological and commercial dead end. The confluence of genetic drift, stochastic gene expression, and metabolic complexity renders the pursuit of absolute determinism in *in vitro* systems not only technically Sisyphean but economically unsustainable (Keurentjes et al., 2011; Majumder et al., 2025; Maranas and Nemhauser, 2025). This impasse, however, contains the seed of its own solution. We must stop asking, “How do we eliminate variability?” and start asking, “How do we harness it?” A fundamental paradigm shift is proposed: reframing inherent variability as a “Discovery Engine.” The very same properties that doomed the old approach—heterogeneity, plasticity, and dynamism—become the drivers for a more powerful strategy: the systematic exploration of chemical space for novel compounds and pathways.

### Strategic perturbation: guiding the chaos for chemical diversity

The first step in this new paradigm is to intentionally and strategically perturb cultures. Instead of using elicitors to maximize the yield of a single known compound, we employ a diverse panel of abiotic and biotic stressors in a “perturb-and-observe” framework (Jain et al., 2024; Zhao et al., 2005). In microbial systems like *Streptomyces*, similar strategies—such as ribosome engineering and high-throughput elicitor screens (HiTES)—have successfully activated silent biosynthetic gene clusters (BGCs) by altering transcriptional machinery or introducing sub-lethal concentrations of antibiotics, respectively (Seyedsayamdost, 2014; Zhu et al., 2019). Applying this logic to plants, we can use elicitors not just for yield, but for chemical discovery. The goal is chemical diversity mining—to provoke the plant’s defensive and regulatory networks into producing a wider array of compounds, including those from silent or cryptic biosynthetic pathways (Ochi, 2017; Scherlach and Hertweck, 2021; Wang et al., 2025).

This approach leverages the plant’s innate plasticity, where environmental cues trigger specific metabolic reconfigurations (Mosoh et al., 2024c; Mosoh and Vendrame, 2025). For instance, the application of methyl jasmonate (MeJA), silver nitrate (AgNO_3_), and polyethylene glycol (PEG) to *Camptotheca acuminata* plantlets elicited the accumulation of camptothecin (CPT) and, crucially, led to the identification of 15 new alkaloids and 25 known CPT analogs (Pu et al., 2022). Similarly, biotic elicitors such as fungal extracts from *Aspergillus niger* or *Botrytis* spp. have been shown to dramatically increase the production of diverse metabolites like gymnemic acid and sanguinarine, often activating pathways that are quiescent under standard culture conditions (Bhaskar et al., 2022). Furthermore, targeted genome editing using CRISPR-Cas9 can now activate specific silent clusters by knocking in strong constitutive promoters, a method proven effective in *Streptomyces* to trigger the production of novel polyketides (Zhang et al., 2017). Adapting such precision activation strategies to plant cell cultures could unlock a vast reservoir of “cryptic” metabolites encoded by the plant genome.

This strategy is made possible by high-throughput, untargeted metabolomics, which acts as a net, capturing the full breadth of the chemical response triggered by these perturbations (Aloo et al., 2023; Tsugawa et al., 2021). By analyzing the “metabolic noise” generated by these perturbations, we can identify novel chemical entities that would otherwise remain hidden in a tightly controlled, uniform culture. Computational tools like DeepRiPP and BiG-SCAPE/CORASON further enhance this process by integrating genomic and metabolomic data to automate the discovery of novel natural products and map their biosynthetic diversity (Merwin et al., 2020; Navarro-Muñoz et al., 2020).

### Single-cell resolution: decoding the metabolic landscape

This guided perturbation creates a complex, heterogeneous population. Crucially, the metabolic “chaos” induced by stress is not a uniform response across the culture; rather, it manifests as high cellular heterogeneity (Li et al., 2025; Stopka et al., 2018). Within an elicited population, only a small fraction of “elite” cells may undergo the specific transcriptional bursting events required to fully activate a target biosynthetic pathway, while the majority remain in low- or non-producing states. Conventional bulk analysis averages these rare, high-flux states with the non-producing majority, diluting the signal and obscuring the underlying regulatory logic (Seth et al., 2025; Wu R. et al., 2024).

To extract value from this “productive chaos,” we must therefore move beyond bulk analysis and zoom in to single-cell resolution. Recent advances in single-cell transcriptomics (scRNA-seq) now allow us to dissect this heterogeneity with unprecedented resolution by profiling thousands of individual cells under stress and computationally isolating rare, high-performing subpopulations (Kang et al., 2025; Wen et al., 2022; Zhu et al., 2025). In parallel, technologies such as Mass Spectrometry Imaging (MALDI-MSI) and Live Single-Cell Mass Spectrometry (LSC-MS) enable the mapping of a culture’s “metabolic landscape,” allowing for the direct visualization and identification of rare, high-producing cells within a population (Myers et al., 2025; Shen et al., 2023).

For example, single-cell metabolomics has revealed significant metabolic heterogeneity even in seemingly uniform biological systems, such as *Catharanthus roseus* leaves, where monoterpene indole alkaloids were found to accumulate specifically in idioblast cells (Pandian et al., 2023). Coupling these metabolite measurements with scRNA-seq provides direct insight into the transcriptional machinery of these elite cells (Nobori, 2025). By correlating single-cell transcriptomes with metabolite profiles, we can uncover the gene regulatory networks driving high production, identifying key transcription factors and biosynthetic enzymes that are diluted or completely lost in bulk datasets (Alam et al., 2025; Pradhan et al., 2025).

This high-resolution, multimodal single-cell data establishes a direct link between a specific metabolic phenotype and its underlying regulatory network. As a result, we are no longer averaging across populations; we are learning from the best performers. Importantly, these data generate high-quality, noise-reduced training datasets that are essential for the AI-driven pathway discovery and optimization engines discussed in the next section (Li C. et al., 2023; Misra et al., 2014).

### Embracing dynamics: time as a dimension of discovery

Chemical discovery is not static. The final strategic shift is to treat time as a dimension of discovery. In the old paradigm, culture aging and subculturing were confounders to be minimized. In the new paradigm, they are experimental variables. By implementing longitudinal multi-omics sampling, we can capture metabolic drift and transient chemical phenotypes—valuable compounds that are only produced for a brief window in the culture’s lifecycle (Petrova and Guler, 2024).

Dynamic gene expression noise, often viewed as detrimental, can drive phenotypic diversity and developmental transitions (Cortijo and Locke, 2020). For instance, single-cell trajectory analysis in *Arabidopsis* roots has revealed developmental progression at high temporal resolution (Nobori, 2025). Applying similar temporal profiling to metabolic output allows us to detect transient accumulation of intermediates or novel end-products that appear only during specific phases of stress response or differentiation (Verma and Shukla, 2015). This longitudinal approach transforms temporal instability from a liability into a rich source of data on pathway regulation and flux (Wang et al., 2022).

Together, these three strategies form a cohesive, technologically enabled pipeline: we perturb to diversify, resolve to understand, and track to capture transient events. This is the operational blueprint for turning “productive chaos” into a discovery engine. Rather than fighting the plant’s inherent tendency towards variability, we use it to access a broader chemical space and deeper biological understanding.

However, this new paradigm generates a deluge of complex, multi-dimensional data. The final, crucial piece of the puzzle is no longer at the bench, but in the server room: a computational toolkit to navigate this complexity. We now turn to the specific technologies that make sense of the chaos, acting as the brain of the discovery engine (Capela et al., 2025; Mutwil, 2020; Navarro-Muñoz et al., 2020). For instance, advanced algorithms like DeepBGC and antiSMASH are essential for identifying and prioritizing biosynthetic gene clusters from these massive datasets, ensuring that the most promising candidates for novel compound production are targeted for further characterization (Greunke et al., 2018; Hannigan et al., 2019).

## Section D: the enabling toolkit—the AI-powered pipeline from chaos to candidate

The strategies in Section C successfully harness biological variability as a discovery engine, but they generate a formidable output: a ‘productive chaos’ of complex chemical data. This deluge of information presents its own challenge—how to find the proverbial needle in a haystack of thousands of compounds. This is where the paradigm shift meets its essential enabler: an AI-powered analytical pipeline that acts as the “mission control” for the entire operation. The purpose of this toolkit is to create a seamless, data-driven pipeline that transforms raw spectral chaos into a prioritized shortlist of novel, bioactive candidate molecules (Chigozie et al., 2025; Mullowney et al., 2023).

### Data acquisition: capturing the high-dimensional chemical universe

The pipeline begins with high-fidelity data acquisition. Advanced hyphenated platforms like LC-Ion Mobility-MS/MS do not merely detect compounds; they separate tricky isomers and generate rich, multi-level fragmentation spectra (MS^n^) (L. Liu et al., 2023; Majeed et al., 2023). Ion mobility spectrometry adds an orthogonal dimension of separation based on collision cross-section (CCS), allowing for the resolution of isomeric structures that co-elute in traditional chromatography and share identical mass-to-charge ratios (Asef et al., 2023). Furthermore, automated data-dependent acquisition (DDA) and data-independent acquisition (DIA) modes, enhanced by AI-driven decision-making on the fly, ensure comprehensive coverage of the metabolome, capturing low-abundance metabolites that would otherwise be lost in the noise (El Boudlali et al., 2025). This captures the full structural complexity and diversity of the elicited cultures, providing the high-resolution raw material for the entire downstream process (Sarkar et al., 2025).

### Data structuring: mapping the metabolic landscape

**Figure 1:**
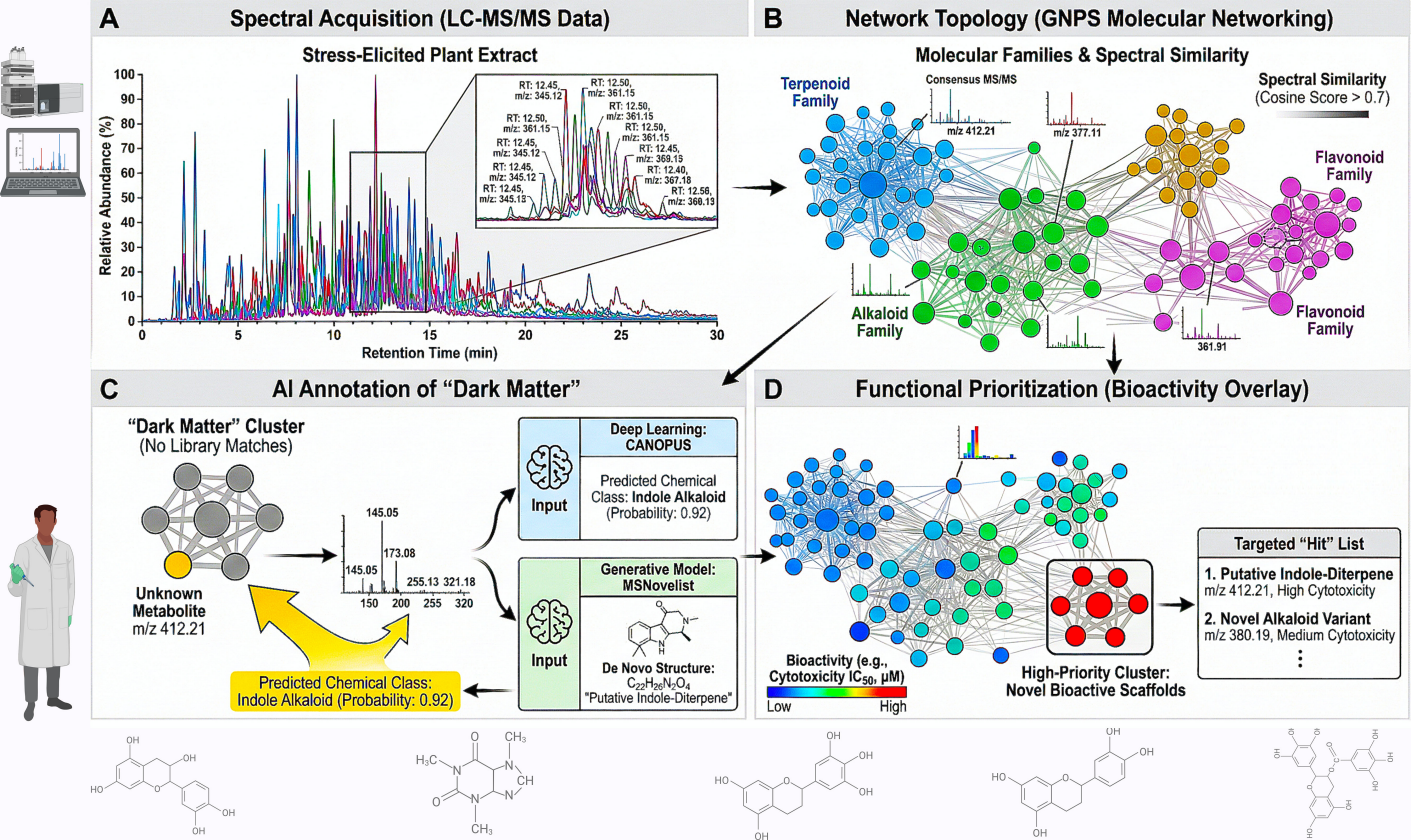
Deconvoluting chemical complexity via AI-guided molecular networking. **(a)** High-resolution liquid chromatography-tandem mass spectrometry (LC-MS/MS) acquires dense spectral data from stress-elicited plant cell cultures, capturing thousands of metabolic features. **(b)** The Global Natural Products Social Molecular Networking (GNPS) platform organizes these spectra based on spectral similarity. Each node represents a consensus MS/MS spectrum; edges connect nodes with high cosine similarity scores (>0.7), clustering structurally related analogues into “molecular families” and revealing metabolic relationships that are invisible in standard chromatograms (Li W. et al., 2023; Selegato et al., 2023). **(c)** Artificial Intelligence-driven annotation tools interrogate “dark matter” nodes (metabolites with no library matches). Deep neural networks (e.g., CANOPUS) predict chemical class (e.g., terpenoid, alkaloid) from fragmentation patterns, while MSNovelist generates *de novo* structural candidates, providing chemical intelligence for unknown metabolites (Dührkop et al., 2021; Stravs et al., 2022). **(d)** Data-driven prioritization is achieved by overlaying phenotypic screening data (e.g., cytotoxicity, fluorescence) onto the network using tools like MolNetEnhancer. This integrates chemotype and phenotype, isolating high-priority clusters (highlighted nodes) that represent novel, bioactive scaffolds for subsequent pathway elucidation and heterologous transfer (Beniddir et al., 2021). This figure was created with the assistance of BioRender (https://biorender.com).

Raw spectral data is fed into computational platforms like Global Natural Products Social Molecular Networking (GNPS) for molecular networking (Zhang M. et al., 2023). This is the crucial organizing step. It visualizes the entire metabolome as an intuitive map, where each molecule is a node and spectral similarity draws the connecting lines (Qin et al., 2022). Recent advancements like Feature-Based Molecular Networking (FBMN) and Ion Identity Molecular Networking (IIMN) have significantly improved the resolution and connectivity of these maps, allowing for the grouping of related adducts and in-source fragments, thereby reducing redundancy and complexity (Schmid et al., 2021; Xiao et al., 2026). Additionally, methods such as mass spectral motif discovery (e.g., MS2LDA) allow for the identification of shared substructures within the network, further illuminating chemical relationships (Beniddir et al., 2021). Instantly, we can see structural families and, most importantly, identify the “dark matter”—dense clusters of molecules with no database matches, which become our prime targets for novelty (Li et al., 2022; Ramabulana et al., 2021). This structured visualization transforms an unintelligible list of features into a navigable chemical space (Baskiyar et al., 2022; Sheng et al., 2024) (**Figure 1**, **Table 1**).

**Table 1:** The AI-powered analytical stack: from spectral data to chemical structure.

Tool category	Software/Algorithm	Primary function	Technical mechanism	Key reference
Molecular Networking	GNPS (Global Natural Products Social Molecular Networking)	Data Organization & Visualization	Organizes MS/MS spectra into clusters based on cosine similarity scores; visualizes chemical families and analogues.	(Wang et al., 2017); (Dührkop et al., 2021); (Schmid et al., 2021)
	FBMN (Feature-Based Molecular Networking)	Quantitative Mapping	Integrates LC-MS feature abundance (retention time/isotope patterns) into the network to differentiate isomers and quantify variants.	(Pakkir Shah et al., 2025); (He et al., 2025); (Selegato et al., 2023); (Xiao et al., 2026)
Chemical Class Prediction	CANOPUS	Class Annotation	Uses deep neural networks (DNN) to predict chemical classes (e.g., “flavonoid,” “terpene”) directly from fragmentation spectra without database matches.	(Dührkop et al., 2021); (Misra, 2021)
	NPClassifier	Class Hierarchy	Classifies natural products into a hierarchical ontology (pathway/superclass/class) linking structure to biosynthesis.	(H. W. Kim et al., 2021)
*De Novo* Structure Elucidation	SIRIUS 4/CSI: FingerID	Fingerprint Prediction	Predicts molecular fingerprints and fragmentation trees from MS/MS data to rank candidate structures from large databases.	(Dührkop et al., 2021); (Dührkop et al., 2019); (Dührkop et al., 2015)
	MSNovelist	Generative Design	Uses generative AI (encoder-decoder neural networks) to predict *de novo* chemical structures for spectra that have no database match (“dark matter”).	(Stravs et al., 2022)
Substructure Discovery	MS2LDA	Motif Discovery	Adapts Latent Dirichlet Allocation (topic modeling) to identify co-occurring mass fragments and neutral losses (Mass2Motifs) representing shared substructures.	(Beniddir et al., 2021); (van Der Hooft et al., 2016)
Bioactivity Linkage	MolNetEnhancer	Data Integration	Integrates output from molecular networking, *in silico* annotation, and substructure mining into a single visualization to link chemotype to phenotype.	(Jarmusch et al., 2021); (Beniddir et al., 2021); (Ernst et al., 2019)

### Intelligent annotation: AI-powered dereplication and prediction

With the map drawn, we need intelligent labels. This is where artificial intelligence takes over. Tools like DEREPLICATOR+ automatically compare thousands of spectra to global databases in minutes, instantly identifying known compounds and saving months of manual dereplication (Du et al., 2024). However, the true power of AI lies in annotating the unknown. For the “dark matter,” tools like CANOPUS and SIRIUS 4 use deep neural networks to predict chemical classes and molecular fingerprints directly from MS/MS spectra, even for compounds never seen before (Dührkop et al., 2021; Wang et al., 2023). Emerging generative models, such as MSNovelist, go a step further by generating *de novo* chemical structures from spectral data, independent of existing databases, thus expanding the search space to truly novel chemical entities (Stravs et al., 2022). Furthermore, integration with Nuclear Magnetic Resonance (NMR) data through AI-enhanced dereplication tools like SMART and MixONat provides complementary structural information, increasing the confidence of annotations (Du et al., 2024). Recent deep learning models, such as MS2DeepScore and Spec2Vec, have further refined spectral similarity scoring, enabling more accurate propagation of annotations across the network (Liu Y. et al., 2025; Russo et al., 2024). These tools provide the first crucial hint about the nature and potential value of unknown metabolites, quantitatively scoring their novelty and chemical plausibility (Cao et al., 2021; Hu and Qiu, 2023; Zhu B. et al., 2024).

### Data-driven prioritization: triaging by function and novelty

The final, decisive step in the discovery pipeline is triage. By integrating data from high-throughput bioassays—targeting either specific protein receptors or broader phenotypic effects such as apoptosis—we can overlay a functional "bioactivity score" onto the established chemical map (Dührkop et al., 2021; Meunier et al., 2024). Computational architectures like NPClassifier and MolNetEnhancer facilitate this by merging chemical classification with bioactivity data, allowing researchers to statistically link specific structural classes to observed biological phenomena (Beniddir et al., 2021; Meijer et al., 2025). Advanced machine learning algorithms have moved beyond simple dereplication, now predicting bioactivity directly from mass spectral features or *in silico* predicted scaffolds, effectively mapping chemotype to phenotype (Brittin et al., 2025; Moshkov et al., 2023). For instance, the integration of untargeted metabolomic profiles with phenotypic screening data enables the identification of specific molecular families responsible for biological activity, allowing for "activity-guided" prioritization in a high-dimensional digital environment (Fredin Haslum et al., 2024; Vincent et al., 2020). This approach transforms discovery from a heuristic search into an objective, data-driven decision-making process, focusing resources on the most promising, novel, and bioactive candidates (Linciano et al., 2023; Pham et al., 2021). Furthermore, predictive tools for biosynthetic gene clusters (BGCs), such as DeepBGC and DeepRiPP, can be synchronized with metabolomic data to prioritize compounds based on their coupled genetic novelty and biosynthetic potential (Hannigan et al., 2019; Merwin et al., 2020).

However, the transition from *in silico* prioritization to therapeutic reality is governed by the principle that bioactivity prediction remains fundamentally probabilistic rather than deterministic. While deep learning architectures have reached impressive accuracy in specific domains, their performance is inherently bounded by the quality, diversity, and latent bias of their training corpora (Caesar et al., 2021; Liao et al., 2025). The predictive fidelity of these models typically exhibits an inverse relationship with structural novelty; as investigations move toward structurally unprecedented metabolites or "dark" chemical space, the lack of representative training data often leads to a precipitous drop in inferential power (Liao et al., 2025).

Furthermore, bioactivity inferred from isolated mass spectral features or static predicted scaffolds often fails to account for the dynamic complexities of physiological environments. Crucial parameters such as bioavailability, metabolic biotransformation, and off-target polypharmacology are frequently absent from current predictive frameworks (Liao et al., 2025; Reinhardt et al., 2025). Within the context of complex biological extracts, observed correlations between molecular families and phenotypic outcomes do not inherently establish causality. Synergistic interactions or indirect metabolic effects within a mixture can confound "activity-guided" algorithms, leading to high false-positive rates (Liao et al., 2025; Singh et al., 2022). Consequently, the contemporary analytical pipeline should be characterized not as a replacement for biochemical rigor, but as a sophisticated hypothesis-generating filter. Computational triage directs limited experimental resources toward high-probability candidates, yet the final validation of the biosynthetic blueprint remains tethered to empirical heterologous expression and rigorous bioassay confirmation (Qin et al., 2025; Wang et al., 2021).

This end-to-end pipeline—from acquisition to prioritization—represents the analytical inflection point that justifies the entire paradigm. It is a systematic, scalable, and intelligent system that decodes the inherent chaos of specialized metabolism, transforming the fundamental problem of biological variability into its greatest strength (Bilbao, 2025; Varghese et al., 2025). By leveraging comprehensive databases and knowledge graphs, such as the Natural Products Atlas and Wikidata, this pipeline not only accelerates discovery but also ensures that findings are integrated into a broader, shared knowledge ecosystem (Meijer et al., 2025; van Der Hooft et al., 2016).

However, identifying a high-priority candidate represents only a partial advance. Once a chemical target has been defined, attention must shift to elucidating the genetic architecture underlying its biosynthesis. A stringent validation framework (**Box 2**) is required to ensure that only fully resolved, functional biosynthetic pathways are selected for downstream transfer. Crucially, these validated genetic blueprints must then be rendered into reliable, scalable outputs, necessitating a production strategy that is insulated from the biological instability inherent to the original discovery host. This requirement motivates the final element of the paradigm: complete decoupling.

Box 2 **The path from peaks to pathways: validating the biosynthetic blueprint**

The transition from identifying mass spectrometry peaks to delineating a complete biosynthetic pathway requires a multi-tiered validation framework that bridges computational predictions with empirical biological reality. This process involves cross-referencing multi-omics associations with biochemical assays and heterologous reconstitution to transform "biosynthetic hypotheses" into "validated blueprints." Crucially, validation is not achieved through correlation alone, but through iterative testing across molecular, genetic, biochemical, and structural levels of evidence.

*1. High-Resolution Metabolite Annotation and Reaction Pairing*

The validation process begins with the structural annotation of mass-spectral features. Tools such as SIRIUS and CSI:FingerID integrate high-resolution isotopepatterns and fragmentation trees to generate ranked predictions of molecular structures (Caesar et al., 2021; Singh et al., 2022). Confidence at this stage depends on orthogonal support, including spectral library matching, in silico fragmentation concordance, and, where feasible, comparison to authentic standards.

 •** Reaction Pair Analysis:** Computational workflows now identify enzymatic reaction pairs by detecting characteristic mass shifts (e.g., ±15.99 Da foroxygenation or ±14.01 Da for methylation) within molecular networks (Reinhardt et al., 2025). When integrated with molecular networking, such mass-difference relationships provide directional hypotheses regarding enzymatic transformations rather than merely structural similarity.

 •** Mass2Motifs:** Algorithms like **MS2LDA** extract "Mass2Motifs"—chemically relevant substructures—allowing researchers to group metabolites sharingcommon core scaffolds even when complete structures are unknown (Caesar et al., 2021; Singh et al., 2022). This substructure-level organization enables scaffold-centric pathway inference, particularly for compound families that resist full structural resolution.

*2. Integrative Gene–Metabolite Association and GRN Inference*

Candidate genes identified via co-expression analysis must be grounded in **Gene Regulatory Networks (GRNs)** to distinguish between direct biosynthetic enzymesand secondary regulatory effects. Moving beyond simple correlation, integrative modeling frameworks link metabolite abundance, gene expression, and regulatory architecture to prioritize causally plausible candidates.

 •** Dynamic Modeling:** Time-series transcriptomics, processed via tools like Dynamic GENIE3 or OutPredict, reveal the temporal order of gene activation,allowing researchers to traverse the regulatory hierarchy from master transcription factors to structural genes (Singh et al., 2022). Temporal precedence strengthens causal inference by aligning enzyme expression with metabolite accumulation trajectories.

 •** Spatial Mapping:** Mass Spectrometry Imaging (MSI) provides a "spatial chemical snapshot," validating that a candidate gene is expressed in the exact tissue orcell type (e.g., glandular trichomes or vascular parenchyma) where the target metabolite accumulates (Zou et al., 2025). Concordant spatial localization of transcripts, proteins, and metabolites offers an additional layer of validation that reduces false-positive gene assignments.

*3. Synthetic Biology as a Definitive Validation Platform*

The gold standard for pathway validation is the ***de novo* reconstitution** of the entire pathway in a heterologous host, typically *Nicotiana benthamiana*, *Saccharomycescerevisiae*, or *Escherichia coli* (Qin et al., 2025; Wang et al., 2021). Functional reconstitution establishes sufficiency by demonstrating that a defined gene set can produce the predicted metabolite outside its native context.

 •** Modular Refactoring:** Complex pathways are often broken into discrete biosynthetic modules. For instance, the vinblastine pathway (30+ steps) was validatedby integrating modules for strictosidine, tabersonine, and catharanthine into yeast (Wang et al., 2025). Stepwise reconstruction enables systematic identification of bottlenecks, cryptic intermediates, and enzyme interdependencies.

 •** Enzyme Characterization:** Refined techniques like **CRISPR-Cas9**-mediated gene editing and **Red/ET recombineering** allow for rapid "swapping" ofsubdomains in assembly-line enzymes (e.g., NRPS or PKS) to confirm substrate specificity and catalytic efficiency (Wang et al., 2025; Wilkinson & Micklefield, 2007). Definitive validation typically requires biochemical assays demonstrating catalytic turnover, kinetic parameters, and, where possible, structural confirmation of products by NMR or high-resolution MS.

*4. AI-Guided Predictive Prioritization*

Artificial Intelligence is increasingly used to fill "missing links" in incomplete pathways. Rather than replacing experimentation, AI frameworks function as hypothesis generators that constrain the search space for empirical testing.

 •** Bio-retrosynthesis:** Tools like **BioNavi-NP** utilize transformer neural networks to predict biologically plausible routes for complex natural products, achievinghigh accuracy in identifying starting building blocks (Liao et al., 2025). Such models rank feasible precursor–enzyme combinations, guiding targeted gene discovery efforts.

 •** Structural Fidelity: AlphaFold2** and **RoseTTAFold** provide high-accuracy 3D protein models, enabling *in silico* docking studies to predict whether a candidateenzyme’s active site can physically accommodate the hypothesized intermediate (Liao et al., 2025; Reinhardt et al., 2025). When coupled with mutagenesis and activity assays, structure-guided predictions can accelerate validation while maintaining experimental rigor.

Together, these layered strategies—spanning computational annotation, network inference, biochemical validation, and synthetic reconstruction—convert correlative multi-omics signals into experimentally substantiated biosynthetic architectures. In this framework, a pathway is considered validated only when structural, genetic, enzymatic, and functional evidence converge on a coherent mechanistic model.

## Section E: a pragmatic blueprint I—engineering an escape from instability

Sections A and B established that the native plant cell is a biologically and commercially unstable production platform. The discovery engine in Sections C and D provides a way to find valuable needles in this haystack. But how do we produce these needles reliably? The answer is a strategic pivot: we must engineer an “escape from instability.” This involves decoupling production from discovery by functionally repatriating the validated biosynthetic pathway into a specialized, industrial microbial host designed for one job: reliable, high-titer production. This approach shifts the challenge from fighting the inherent volatility of plant biology to mastering the predictable control of microbial engineering (Ke and Yoshikuni, 2020; Liu et al., 2017).

### Strategic chassis selection: the “fit-for-purpose” host

The first decision is the most critical: choosing the production host. Instead of forcing pathways into standard lab workhorses like *Escherichia coli* or *Saccharomyces cerevisiae*, we now strategically select a “fit-for-purpose” chassis that offers an innate metabolic advantage. This “metabolic context matching” provides a crucial head start by leveraging a host’s native machinery to support the specific demands of the target pathway (Liu J. et al., 2020; Xu et al., 2020a).

For example, the oleaginous yeast *Yarrowia lipolytica* has emerged as a superior host for terpenoid production due to its high flux through the mevalonate pathway and abundant supply of acetyl-CoA, a key precursor (Ma et al., 2021; Zhang T.-L. et al., 2023). Similarly, *Streptomyces* species, with their native capacity for polyketide and non-ribosomal peptide synthesis, are ideal for producing complex secondary metabolites that require specialized post-translational modifications (Del Carratore et al., 2022; Liu et al., 2018). Moreover, recent work has demonstrated that non-conventional hosts like *Pichia pastoris* and *Corynebacterium glutamicum* offer unique advantages for specific classes of compounds, such as high-level protein expression and robust amino acid metabolism, respectively (Gao et al., 2024; Xu et al., 2022). By selecting a host that is metabolically predisposed to the target molecule, we minimize the need for extensive foundational engineering and reduce the metabolic burden on the cell.

### Maximizing stability and yield: taming metabolic burden

Once the host is selected, the pathway must be installed for the long haul. This means moving beyond fragile plasmids, which are prone to segregation instability and copy number variation, to stable genomic integration. Genomic integration ensures consistent gene dosage and expression levels across generations, a prerequisite for industrial scale-up (Chu, 2022; Xu et al., 2022). Advanced genome editing tools, such as CRISPR-Cas9 and transposon-based systems, have revolutionized this process, enabling precise, multiplexed integration of large biosynthetic gene clusters into stable genomic loci (Kim et al., 2025; Wang et al., 2018). Innovative tools like Direct Pathway Cloning (DiPaC) further streamline the capture and refactoring of large biosynthetic gene clusters, facilitating their stable integration into heterologous hosts (Greunke et al., 2018).

However, stable expression alone is not enough. High-level production of heterologous enzymes and metabolites often imposes a significant metabolic burden, leading to growth retardation and genetic instability. To “tame” this burden, we must employ dynamic control strategies. By using metabolite-responsive biosensors or optogenetic switches to decouple growth from production, we can delay pathway activation until the host has reached sufficient biomass, thereby maximizing both titer and productivity (Wang et al., 2021; Yang et al., 2022). For instance, dynamic regulation of the shikimate pathway in *Corynebacterium glutamicum* using a *p*-coumaric acid-responsive biosensor significantly improved the production of aromatic compounds by balancing precursor supply with cell growth (Liu et al., 2017). Additionally, ribosome engineering has emerged as a powerful strategy to activate silent gene clusters and enhance secondary metabolite production, as demonstrated in *Streptomyces* species (Ochi, 2017; Xu et al., 2022; Zhu et al., 2019).

### Advanced cellular architecture: compartmentalization for efficiency

The final refinement involves optimizing the host’s internal architecture by repurposing eukaryotic organelles as specialized, orthogonal bioreactors. Eukaryotic cells offer a unique advantage here: membrane-bound organelles. By compartmentalizing pathways within organelles like peroxisomes, mitochondria, or lipid droplets, we can create specialized bioreactors within the cell (Du and Li, 2021; Jin et al., 2022). This strategy goes beyond simple enzyme localization; it requires the holistic engineering of the organelle’s physicochemical environment to support high-flux biosynthesis (Du and Li, 2021; Jin et al., 2022).

By sequestering pathways within membrane-bound compartments, we achieve three distinct advantages: the concentration of intermediates and enzymes to enhance catalytic efficiency, the physical isolation of toxic products or intermediates from the cytosol, and the protection of the engineered pathway from competing reactions and native cytosolic regulation (Chen et al., 2023; Schenck and Last, 2020).

In yeast, for example, targeting the mevalonate pathway and terpene synthases to the peroxisome has been shown to dramatically increase the production of squalene and other terpenoids by harnessing the organelle’s high acetyl-CoA pool and isolating the pathway from cytosolic regulation (Kulagina et al., 2021; Liu H. et al., 2020).

However, sequestration also creates new metabolic demands. A confined pathway can rapidly deplete an organelle’s native cofactor pool, making successful architectural engineering dependent on a systems-level redesign of the organelle itself. This includes upregulating specific transporters to maintain precursor and metabolite flux and engineering auxiliary enzymes to regenerate critical cofactors such as ATP and NADPH *in situ* (Liu J. et al., 2020). Without these supporting modifications, compartmentalization can shift, rather than resolve, metabolic bottlenecks.

For lipophilic compounds, the properties and capacity of the compartment itself often become rate-limiting. Recent advancements have demonstrated the utility of targeting enzymes to lipid droplets for the production of hydrophobic metabolites, such as ginseng saponins, where the droplets provide a suitable nonpolar environment that significantly boosts yields (Shi Y. et al., 2021). Furthermore, engineering lipid droplets to serve as expandable storage depots for compounds like α-bisabolene can alleviate product toxicity and drive reaction equilibrium toward synthesis, enabling titers that would be impossible in a non-compartmentalized system (Lu Z. et al., 2023; Zhou et al., 2025). This approach effectively creates a “metabolic sink” for the final product, continuously pulling flux through the pathway and protecting the cell from feedback inhibition (Lu J. et al., 2023; Ma et al., 2021).

Crucially, the functional expression of plant cytochrome P450s—the enzymes responsible for the complex oxidations that define many high-value secondary metabolites—requires specific architectural interventions to support this compartmentalization. In native plant tissues, these enzymes are anchored to the endoplasmic reticulum (ER), but in microbial hosts, this localization is often inefficient. To overcome this, strategies such as N-terminal truncation or replacement with soluble tags (e.g., the 8RP peptide) have proven effective in improving solubility and preventing aggregation in *E. coli* and yeast (Biggs et al., 2016; Poborsky et al., 2023). Furthermore, the stoichiometry between the P450 and its redox partner, Cytochrome P450 Reductase (CPR), must be carefully tuned; an excess of CPR often leads to electron uncoupling and toxic Reactive Oxygen Species (ROS) generation. Recent work has demonstrated that a high P450:CPR ratio favors efficient coupling and product formation (Cha et al., 2022; Sun et al., 2023). Finally, the physical capacity of the host’s membrane system can be engineered. In *Saccharomyces cerevisiae*, overexpression of the ER-regulator INO2 triggers the expansion of the ER membrane, significantly increasing the folding capacity and accommodation space for membrane-bound P450s, thereby boosting titers of complex terpenoids (Kim et al., 2019; Wu et al., 2024).

Beyond natural organelles, the development of artificial organelles and membrane-less compartments through liquid–liquid phase separation offers even more sophisticated control over enzyme colocalization, local metabolite concentrations, and metabolic flux (Chen et al., 2023). Together, these strategies illustrate how advanced cellular architecture, when coupled with systems-level organelle engineering, can transform eukaryotic cells into highly efficient, modular platforms for complex biosynthesis (**Box 3**, **Box 4**). Together, these steps allow us to construct a dedicated production organism—a biological factory insulated from the genetic drift, epigenetic noise, and metabolic complexity of the plant cell. This architectural and systems-level foundation is the cornerstone of a reliable biomanufacturing process.

Box 3 **Engineering subcellular architecture: peroxisomes and lipid droplets as synthetic bioreactors for high-flux terpenoid production**

The evolution of microbial cell factories has progressed from linear pathway overexpression to deliberate manipulation of cellular architecture. This transition is particularly evident in the biosynthesis of complex isoprenoids such as squalene, α-farnesene, and α-bisabolene, whose production is constrained by the need for exceptionally high fluxes of acetyl-CoA, NADPH, and ATP, as well as by severe cytotoxicity arising from the accumulation of hydrophobic intermediates and end products. A growing body of evidence demonstrates that subcellular compartmentalization—specifically the repurposing of eukaryotic organelles as orthogonal bioreactors—represents a central paradigm for overcoming these limitations (Du and Li, 2021; Jin et al., 2022; Kulagina et al., 2021; Tran and Lee, 2025).

***Peroxisomes as Flux Concentrators and Autonomous Production Units***

Among intracellular compartments, the peroxisome has emerged as a premier chassis for acetyl-CoA–derived terpenoid biosynthesis. Peroxisomes naturally generate high local concentrations of acetyl-CoA via β-oxidation and are physically insulated from cytosolic regulatory networks, enabling the concentration of enzymes and substrates while sequestering toxic intermediates (Kulagina et al., 2021). However, effective peroxisomal engineering requires not only precursor supply but also dedicated cofactor regeneration and energy transport, as the organelle membrane is largely impermeable to cytosolic acetyl-CoA and redox equivalents.

Early demonstrations of this principle showed that targeting the mevalonate (MVA) pathway to the peroxisome in *Yarrowia lipolytica* substantially increased squalene production when coupled with cofactor engineering. Liu H. et al. (2020) transformed the peroxisome into a high-flux synthetic organelle by overexpressing peroxisomal NADP^+^-dependent isocitrate dehydrogenases (IDP2 and IDP3) to regenerate NADPH and the ATP transporter ANT1 to sustain energy balance, resulting in a 28-fold increase in squalene titer to 502.7 mg/L (Liu H. et al., 2020). These results established that peroxisomal autonomy—defined by balanced precursor, redox, and energy supply—is a prerequisite for robust biosynthesis.

Subsequent work has pushed this strategy to industrially relevant scales. Ning et al. (2024) implemented a dual cytoplasmic–peroxisomal architecture in *Y. lipolytica*, in which cytosolic overexpression of rate-limiting enzymes (tHMG1, IDI1, ERG9) was combined with complete relocalization of the MVA pathway to the peroxisome. A key technical advance was the identification of a highly efficient peroxisomal targeting signal (CRMVGKSKL), which outperformed the classical SKL motif and increased squalene titers by 12.3% (Ning et al., 2024). By simultaneously upregulating β-oxidation genes (POT1 and POX1) to drive intra-peroxisomal acetyl-CoA generation, this design yielded a record squalene titer of 51.2 g/L in a 5-L bioreactor, corresponding to 60.5 mg/g glucose (Ning et al., 2024). These findings align with broader analyses showing that peroxisomal compartmentalization can enhance monoterpene and sesquiterpene production by up to 125-fold relative to cytosolic expression (Kulagina et al., 2021).

Beyond catalysis, peroxisomes also function as dynamic storage depots for lipophilic products. Using laser scanning confocal microscopy and transmission electron microscopy, Liu G. S. et al. (2020) demonstrated that squalene accumulation induces peroxisomal inflation into lipid-droplet-like structures, revealing an intrinsic capacity of the peroxisomal matrix to sequester hydrophobic compounds. This dual role—simultaneous synthesis and storage—mitigates cytotoxicity and overcomes the limited storage capacity of cytosolic lipid droplets (Liu G. S. et al., 2020). Exploiting this property, hybrid “dual cytoplasmic–peroxisomal” strains generated through haploid mating achieved 11.0 g/L squalene in two-stage fed-batch fermentation (Liu G. S. et al., 2020).

***Lipid Droplets as Hydrophobic Sinks and Equilibrium Drivers***

While peroxisomes act as flux concentrators, lipid droplets (LDs) serve as specialized sinks for lipophilic toxicity. Many terpenoids readily partition into cellular membranes, disrupting integrity and inhibiting growth. LDs, composed of a neutral lipid core surrounded by a phospholipid monolayer, provide a hydrophobic reservoir that protects the cell while thermodynamically pulling biosynthetic reactions forward. By tethering pathway enzymes to LD-surface proteins such as perilipins or oleosins, engineers can spatially couple synthesis with immediate sequestration.

A striking example is the “push–pull” strategy developed by Lu Z. et al. (2023) for α-bisabolene production in *Y. lipolytica*. Upregulation of the MVA pathway (“push”) was paired with deliberate expansion of LD storage capacity (“pull”) via overexpression of diacylglycerol acyltransferase (DGA1) and fatty acid desaturase (OLE1). The enlarged LD pool alleviated cytotoxicity and shifted reaction equilibrium toward product formation, resulting in a record α-bisabolene titer of 1954.3 mg/L—a 96-fold improvement over the control strain (Lu Z. et al., 2023). Similar LD-expansion strategies have been shown to enhance squalene accumulation by creating a robust intracellular sink for lipophilic products (Chai et al., 2024; Tran and Lee, 2025).

***Cofactor, Organelle Crosstalk, and Secretion Engineering***

High-flux compartmentalized biosynthesis is ultimately constrained by cofactor availability. Multiple studies demonstrate that redox and energy balance must be engineered at the whole-cell level to support organelle-localized pathways. In *Komagataella phaffii*, Fina et al. (2025) showed that overexpression of a cytosolic–peroxisomal redox shuttle (IDP2/IDP3) restored peroxisomal NADPH pools and significantly improved pathway efficiency (Fina et al., 2025). Complementary strategies in *Y. lipolytica*, including NADPH recycling via mannitol dehydrogenase (ylMnDH2), further underscore the importance of cofactor regeneration (Liu H. et al., 2020).

Even with expanded intracellular storage, saturation can occur. To overcome this ultimate bottleneck, secretion engineering has emerged as a critical extension of cellular architecture design. Chai et al. (2025) developed a carrier protein–mediated trafficking system in *Y. lipolytica* by fusing the lipid-binding domain of the oxysterol-binding protein OSH3 with a secretion signal peptide. Coupled with the ABC transporter SNQ2, this system exported 3.43 g/L of squalene extracellularly—27.2% of total production—representing the highest reported extracellular squalene titer and effectively bypassing intracellular storage limits (Chai et al., 2025).

***Conceptual Synthesis***

Collectively, these studies demonstrate that advanced cellular architecture is not a single intervention but a synergistic design philosophy. Peroxisomes function as autonomous, high-flux production hubs; lipid droplets act as hydrophobic sinks that relieve toxicity and drive equilibrium; and cofactor engineering and secretion systems integrate these compartments into a coherent, highly productive cellular factory. Through this compartmentalized framework, terpenoid titers have been elevated from milligram-per-liter levels to beyond 50 g/L, marking a fundamental shift in the limits of microbial isoprenoid biosynthesis (Ning et al., 2024).

Box 4 **Mitochondria and the endoplasmic reticulum as enabling organelles in compartmentalized terpenoid biosynthesis**

While peroxisomes and lipid droplets are increasingly recognized as the primary catalytic and storage bioreactors for high-flux terpenoid synthesis, mitochondria and the endoplasmic reticulum (ER) play indispensable enabling roles that sustain redox balance, energy supply, enzyme functionality, and downstream product handling. Rather than acting as dominant sites of carbon flux, these organelles function as auxiliary yet essential modules that stabilize, amplify, and extend the performance of compartmentalized biosynthetic architectures (Kulagina et al., 2021; Tran and Lee, 2025).

***Mitochondria: Redox and Precursor Powerhouses Supporting Compartmentalized Flux***

Mitochondria possess inherently high concentrations of acetyl-CoA and ATP, along with segregated pools of redox cofactors, making them attractive targets for metabolic engineering. However, their application in terpenoid biosynthesis is constrained by the impermeability of the inner mitochondrial membrane to phosphorylated intermediates and limited metabolite exchange with the cytosol and peroxisomes (Tran and Lee, 2025). As a result, contemporary strategies emphasize *redox and energy* coupling rather than wholesale pathway relocalization.

Redox shuttle engineering has emerged as a central solution to these constraints. Fina et al. (2025) demonstrated in *Komagataella phaffii* that co-overexpression of a cytosolic NADH kinase (cPos5) with a mitochondrial NADPH-dependent isocitrate dehydrogenase shuttle (Idp2/Idp3) effectively linked cytosolic NADH generation to mitochondrial NADPH regeneration. Using 3-hydroxypropionic acid as a reporter metabolite, this study validated that engineered redox coupling can drive mitochondrial biosynthetic reactions, effectively transforming the organelle into a chemically integrated yet spatially distinct production unit (Fina et al., 2025).

Importantly, this work established that mitochondrial engineering need not rely on direct pathway insertion to be impactful. By optimizing the flux of reducing equivalents across organelle boundaries, mitochondria can function as tunable redox reservoirs that indirectly enhance acetyl-CoA–derived anabolic pathways, including terpenoid biosynthesis. These findings are consistent with broader analyses showing that mitochondrial redox rewiring can alleviate global cofactor bottlenecks and improve cytosolic and peroxisomal productivity (Kulagina et al., 2021).

***Endoplasmic Reticulum: Functionalization, Folding, and Surface Engineering***

The ER occupies a distinct and non-redundant niche in terpenoid biosynthesis as the primary site for membrane-bound cytochrome P450 monooxygenases and other tailoring enzymes required for terpene functionalization. Unlike peroxisomes, the ER is not optimized for high precursor flux; instead, it provides a specialized lipid and protein-folding microenvironment essential for enzymatic specificity, stability, and electron transfer.

To overcome the intrinsic limitations of ER capacity, architectural engineering strategies have focused on membrane expansion. Wang et al. (2025) demonstrated that overexpression of the transcriptional regulator INO2 or deletion of the phosphatidate phosphatase PAH1 induces extensive ER membrane proliferation. This physical expansion generates a favorable microenvironment that reduces enzyme aggregation, enhances coupling between P450s and their cytochrome P450 reductase partners, and alleviates ER stress (Wang et al., 2025). The result is a marked increase in catalytic efficiency and titers of functionalized terpenoids, underscoring that organelle morphology and surface area are as critical as enzyme expression levels.

Beyond enzyme performance, the ER also serves as a central hub for intracellular trafficking and secretion of hydrophobic metabolites. Vesicle-mediated transport originating from the ER has been exploited to relieve intracellular accumulation limits. Chai et al. (2025) engineered an ER-associated carrier protein–mediated trafficking system by fusing the lipid-binding domain of the oxysterol-binding protein OSH3 with a secretion signal peptide. When combined with the ABC transporter SNQ2, this ER-centered export module enabled extracellular accumulation of 3.43 g/L squalene—27.2% of total production—representing the highest reported extracellular squalene titer and effectively bypassing intracellular storage saturation (Chai et al., 2025).

***Functional Integration within Multi-Organelle Architectures***

Collectively, mitochondrial and ER engineering does not supplant peroxisomal or lipid droplet–based bioreactors but instead integrates with them to form a hierarchical, multi-organelle production system. In this architecture, peroxisomes serve as high-flux synthesis hubs, lipid droplets buffer hydrophobic toxicity, mitochondria stabilize redox and energy balance, and the ER ensures efficient enzyme folding, functionalization, and product trafficking. This division of labor reflects a broader shift from pathway-centric optimization toward holistic cellular design, in which organelles are treated as interoperable modules within a compartmentalized metabolic factory (Kulagina et al., 2021; Tran and Lee, 2025).

By incorporating mitochondria and the ER as enabling—rather than primary—production compartments, metabolic engineers have substantially expanded the operational envelope of microbial terpenoid biosynthesis, enabling sustained high titers, improved robustness, and scalable performance across diverse hosts and cultivation regimes.

However, designing and optimizing these multi-step pathways within a new host is a non-trivial challenge. It requires balancing flux, matching enzyme kinetics, and avoiding toxic intermediates—a multi-dimensional optimization problem that quickly exceeds human intuition. Moreover, for pathways where the full enzymatic complexity remains uncharacterized or requires tissue-specific compartmentalization that cannot yet be replicated in microbes, differentiated plant tissues such as hairy roots offer a genetically stable, albeit less scalable, alternative (**Box 5**). This divergence in platform choice underscores the need for rational design principles and predictive tools, and leads us to the second part of our blueprint, where we leverage the power of computation to navigate this design space.

Box 5 **The hairy root exception: when differentiated plant tissue is the superior chassis**

While microbial fermentation offers the highest ceiling for scalability, it is not the only solution to the instability of plant cell culture. Hairy root cultures, generated by infection with *Agrobacterium rhizogenes*, represent a unique “middle ground”—a differentiated plant tissue that grows in contained liquid culture (Gantait and Mukherjee, 2021). Unlike dedifferentiated cell suspensions, hairy roots maintain a high degree of genetic and biosynthetic stability over long periods, as their chromosomal integrity is preserved by the organized tissue structure (Zhu Y. et al., 2024).

This chassis is particularly advantageous for:

Complex, Root-Specific Pathways: Metabolites like ginsenosides and tropane alkaloids (e.g., scopolamine), which rely on root-specific organelles and transporters that are difficult to reconstruct in yeast or *E. coli* (Aghaali et al., 2024; Shi M. et al., 2021).

“Orphan” Pathways: For bioactive compounds where the complete enzymatic pathway is unknown, hairy roots provide a native environment that naturally expresses the necessary machinery, bypassing the need for full pathway elucidation required for microbial engineering (Mirmazloum et al., 2024).

However, hairy roots face their own “scaling ceiling.” Their complex, branching morphology creates rheological challenges in standard bioreactors, leading to mass transfer limitations and shear stress sensitivity that are less problematic in unicellular microbial systems (Morey and Peebles, 2022; Srivastava and Srivastava, 2007). Thus, while hairy roots are a powerful tool for producing complex, root-derived metabolites that defy microbial synthesis, they serve as a specialized “Plan B” chassis rather than a universal replacement for the scalability of microbial fermentation (Liu C. et al., 2025).

## Section F: a pragmatic blueprint II—closing the loop with predictive computational design

Section E provided the stable production vessel, but optimizing the complex biochemical pathways within it has traditionally been a slow, iterative process of trial and error. We now close the loop with the final piece of the blueprint: a predictive computational layer that acts as the central intelligence for the entire pipeline. The same AI revolution that powers our discovery engine now drives the rational design of our production system. This creates a continuous “design-build-test-learn” cycle, where data from the engineered host is used to fuel models that predict the next, most effective engineering intervention, drastically reducing development time and cost (Choi et al., 2019; Helmy et al., 2020). Recent advances demonstrate that AI can not only predict outcomes but also autonomously design and execute experiments, moving towards self-driving laboratories that integrate biofoundries with predictive modelling (Chen Q. et al., 2025; Groff-Vindman et al., 2025).

### Multi-omics driven debugging: diagnosing the engineered host

When a heterologous pathway underperforms, the first question is “why?” Modern multi-omics provides the answer. By simultaneously analyzing the transcriptome, proteome, and metabolome of the engineered host, we can diagnose the failure mode with high resolution. Is the mRNA not being transcribed? Are the enzymes being degraded? Is a key precursor being diverted? This systems-level diagnosis moves us from guessing to knowing.

For instance, integrated multi-omics analysis has been successfully used to identify regulatory bottlenecks in *Escherichia coli* strains engineered for biofuel production, revealing non-obvious targets for genetic intervention that significantly increased titers (Costello and Martin, 2018). Similarly, in *Saccharomyces cerevisiae*, time-series multi-omics data combined with machine learning uncovered dynamic pathway limitations that were invisible to static analysis, guiding the implementation of dynamic control strategies (Roy et al., 2021). Tools like INTEGRATE further enhance this capability by using constraint-based modeling to distinguish between transcriptional and metabolic regulation, enabling precise targeting of the underlying cause of flux imbalance (Di Filippo et al., 2022). Moreover, the integration of machine learning with multi-omics data allows for the identification of non-intuitive regulatory relationships and the prediction of pathway dynamics from time-series data, providing a robust framework for debugging engineered strains (Roy et al., 2021; Zampieri et al., 2019).

### Predictive modeling of pathway bottlenecks

With a diagnosed problem, we can predict the solution. Genome-scale metabolic models (GEMs) and machine learning algorithms can simulate the entire host’s metabolism. They allow us to run *in silico* experiments, predicting which genetic modifications will re-route flux toward our target compound and which will simply cripple the host. We can prototype thousands of strain designs computationally before ever touching a “pipette”.

Recent advances in constraint-based modeling have demonstrated remarkable predictive power in identifying non-intuitive bottlenecks that extend beyond simple carbon flux (Wu et al., 2021; Zampieri et al., 2019).

In particular, genome-scale metabolic models (GEMs) enable the global balancing of cofactor pools—specifically ATP and NADPH—which are often the true limiting reagents in high-flux engineered pathways rather than carbon itself (Ding et al., 2024; Mao et al., 2024). This capability is critical for the rational design of compartmentalized and high-yield production strains.

For example, the dramatic 28-fold increase in squalene production achieved via peroxisomal sequestration (**Box 3**) required not just pathway localization, but the predictive engineering of the peroxisomal cofactor supply. Specifically, this involved the overexpression of *IDP2/IDP3* to boost NADPH generation and *ANT1* to enhance ATP transport into the organelle (Liu H. et al., 2020; Lu J. et al., 2023). Without predictive models to identify these stoichiometric and energetic deficits, compartmentalization strategies often fail due to severe energy or redox imbalances (Mu et al., 2024; Smith et al., 2024).

Beyond classical flux balance analysis (FBA), hybrid models that integrate mechanistic constraints with data-driven predictions have further expanded the design space. These approaches can identify complex, non-intuitive knockout targets and optimize flux distribution and redox states, as demonstrated in the enhanced production of compounds such as ethanol and succinic acid (Wu D. et al., 2024; Zhang et al., 2025). Machine learning algorithms trained on large datasets of strain performance can predict the outcomes of genetic modifications with high accuracy, enabling the rapid *in silico* screening of potential designs before experimental implementation (Kim et al., 2020; Presnell and Alper, 2019).

Beyond flux optimization, deep learning approaches have been applied to predict enzyme turnover numbers and kinetic parameters, filling critical gaps in metabolic reconstructions and enabling more accurate dynamic simulations of engineered pathways (Costello and Martin, 2018; Heckmann et al., 2018). Tools such as BioAutomata and ART exemplify this predictive capability, leveraging Bayesian optimization and ensemble modeling to recommend optimal genetic designs and process conditions (Roy et al., 2021; Volk et al., 2020, 2023). Collectively, these predictive tools not only accelerate strain engineering but also de-risk the scale-up process by identifying potential metabolic instabilities early in the design phase, before they manifest as costly failures in production.

### AI-guided protein and pathway (re)design

For the most stubborn bottlenecks, we can now redesign the molecular components themselves. Tools like AlphaFold2 provide high-confidence protein structures, enabling rational engineering of enzymes for better stability, activity, or substrate specificity (Noordijk et al., 2024; Yang et al., 2019). Looking forward, generative AI can propose entirely novel enzyme sequences or even non-native biochemical pathways to synthesize the target molecule more efficiently. This is the frontier of predictive design.

**Figure 2:**
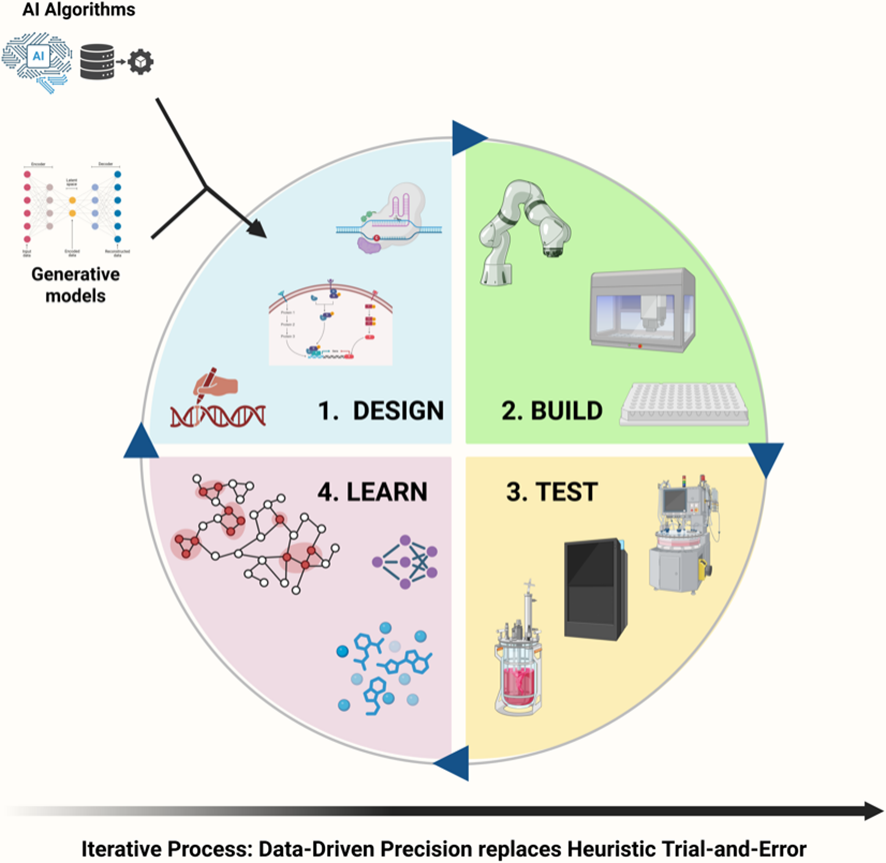
The AI-Driven Design-Build-Test-Learn (DBTL) loop for strain optimization. The cycle integrates predictive computation with automated experimentation to accelerate strain optimization. (1) Design: AI algorithms (e.g., BioAutomata, ART) propose genetic modifications (promoter tuning, gene knockouts) based on prior data or probabilistic models (Roy et al., 2021; Volk et al., 2020). Generative models (e.g., VAEs, GANs) design novel enzyme sequences or pathways (Repecka et al., 2021). (2) Build: Biofoundries utilizing automated liquid handling and CRISPR-based multiplex editing (e.g., CREATE) construct thousands of variant strains in parallel (Abbate et al., 2023; Chen J. et al., 2025). (3) Test: High-throughput phenotyping via micro-bioreactors and multi-omics analysis (transcriptomics, proteomics, metabolomics) generates dense performance datasets (Marcellin and Nielsen, 2018). (4) Learn: Machine learning models trained on multi-omics data identify non-intuitive bottlenecks (e.g., kinetic limitations vs. transcriptional regulation) and update the design algorithms, closing the loop (Costello and Martin, 2018; Di Filippo et al., 2022). This iterative process replaces heuristic trial-and-error with data-driven precision. This figure was created with the assistance of BioRender (https://biorender.com).

Generative models, such as variational autoencoders (VAEs) and generative adversarial networks (GANs), are being used to explore vast protein sequence spaces, generating novel variants with improved properties that are unreachable by traditional directed evolution (Mardikoraem et al., 2023; Notin, 2023; Repecka et al., 2021). In pathway design, tools like BioNavi-NP and RetroPath RL leverage deep learning and reinforcement learning to propose retrosynthetic routes that bypass native regulatory controls or utilize alternative precursors, effectively “short-circuiting” metabolic bottlenecks (Gricourt et al., 2024; Xie et al., 2024; Zheng S. et al., 2022). AI-driven platforms like MassKG combine knowledge-based fragmentation with deep learning to annotate mass spectra and discover novel natural product structures, further bridging the gap between discovery and design (Zhu B. et al., 2024). Additionally, machine learning-guided directed evolution allows for the efficient navigation of fitness landscapes, optimizing enzyme function with fewer experimental iterations (Kouba et al., 2023; Xu et al., 2020b). These AI-driven design capabilities are transforming metabolic engineering from a descriptive science into a predictive, constructive discipline (**Figure 2**, **Table 2**).

**Table 2:** Machine learning approaches for metabolic pathway and enzyme optimization.

Application domain	Machine-learning approach	Principle	Representative outcome	Key references
Pathway Optimization	Bayesian Optimization (e.g., BioAutomata)	Probabilistic exploration of multidimensional design spaces (e.g., promoters, ribosome-binding sites (RBSs)) to identify optimal expression configurations while minimizing experimental iterations.	Optimization of the lycopene pathway in *E. coli* by screening <1% of possible variants, identifying global optima not accessible through random or exhaustive search.	(Chen X. et al., 2019; HamediRad et al., 2019)
Metabolic Debugging	Ensemble Modeling (e.g., ART (Automated Recommendation Tool))	Integrates multiple predictive models (e.g., neural networks, random forests) to correlate multi-omics datasets with metabolite production and recommend genetic interventions.	Increased isoprenol production by 23% through prediction of non-intuitive combinatorial genetic modifications derived from synthetic multi-omics training data.	(Roy et al., 2021);
Enzyme Engineering	Deep Learning (e.g., UniRep (Unified Representation), ESM (Evolutionary Scale Modeling)	Learns high-dimensional protein sequence representations from large unlabelled datasets to predict variant fitness, stability, and functional properties.	Identification of stability- and activity-enhancing mutations without structural input, enabling zero-shot prediction of protein fitness landscapes.	(Yang et al., 2019); (Alley et al., 2019)
Flux Prediction	Hybrid ML-Mechanistic Models	Integrates flux balance analysis (FBA) with machine learning to constrain metabolic solution spaces using experimental data and improve predictive accuracy.	Improved prediction of ethanol and succinic acid production in *S. cerevisiae* relative to FBA alone; identification of novel gene knockout targets.	(Wu D. et al., 2024); (Zampieri et al., 2019)
Retrosynthesis	Reinforcement Learning (e.g., RetroPath RL, BioNavi-NP)	Explores large biochemical reaction spaces to propose de novo biosynthetic routes by iteratively optimizing reaction policies learned from curated databases.	Proposed plausible biosynthetic pathways for structurally complex natural products by integrating reaction rules and data-driven policy learning.	(Xie et al., 2024); (Zheng S. et al., 2022)

This computational layer is the glue that binds the paradigm together. The AI that helps us find a novel compound in a chaotic plant extract is now the same intelligence that helps us optimize its production in a stable microbial host. We have moved from fighting biological complexity to leveraging it for discovery and then using computational power to impose elegant, efficient design for production.

This fully articulated, computationally-driven paradigm—from embracing biological variability for discovery to employing predictive design for stable production—does more than just solve technical problems; it establishes a new, robust, and accelerated R&D pipeline with the power to revitalize natural product discovery and redefine its economic model, a perspective developed in the concluding section.

## Section G: navigating the path to market—the regulatory and commercial virtues of a defined system

The scientific and technological case for this new paradigm is compelling. However, for a true transformation of the field, it must also prove superior where it matters most: in the boardroom and the regulatory agency. The decoupled model doesn’t just make better science; it makes smarter business and a clearer path to the patient. By replacing an unpredictable biological source with a defined manufacturing platform, we systematically de-risk the most costly and uncertain phases of drug development: manufacturing and regulatory approval.

### Regulatory de-risking through a defined production platform

Regulators demand consistency. The old paradigm of plant cell culture fights a losing battle against intrinsic variability, leading to a mountain of Chemistry, Manufacturing, and Controls (CMC) data to prove batch equivalence. Natural extracts, by definition, are complex mixtures subject to seasonal and geographical variations, making it notoriously difficult to meet the stringent purity and consistency standards required for pharmaceutical approval (Tegtmeier et al., 2023; Uhl et al., 2023). This framework is predicated on the use of a defined heterologous host in a controlled fermentation process from the outset. This is “Quality by Design” (QbD), providing the consistent, well-characterized Active Pharmaceutical Ingredient (API) that regulators require, dramatically simplifying the approval dossier (Elder, 2017; Raghavan and McCombie, 2017).

Regulatory bodies like the FDA (U.S. Food and Drug Administration) and EMA (European Medicines Agency) have established clear pathways for biologics and small molecules produced via microbial fermentation, offering a predictable route to market that contrasts sharply with the regulatory hurdles faced by complex botanical mixtures (Cockroft and Wilson, 2021; Sahoo et al., 2009). By adopting a defined production platform, we align with established regulatory frameworks, reducing the risk of delays and rejections associated with variability and impurities (Uhlenbrock et al., 2018). This shift from empirical observation to engineered control ensures that every batch meets the same rigorous specifications, a prerequisite for modern pharmaceutical manufacturing (Davies, 2010).

### A fortified intellectual property position

Commercially, the model creates a formidable intellectual property (IP) position. The discovery engine generates valuable “composition of matter” patents on novel molecules, which are the bedrock of pharmaceutical value (Fialho and Chakrabarty, 2012). But the real, long-term value lies in the process patents for the engineered production system. This creates a dual-layer defense: even if a molecule’s structure patent is challenged, the efficient, proprietary manufacturing process remains a protected and valuable asset (Chakrabarty, 2010; Gurgula, 2017).

The ability to patent the specific genetic modifications, metabolic pathways, and fermentation conditions used to produce a compound adds a robust layer of protection that is difficult for competitors to replicate (David et al., 2015; Sharma et al., 2026). This “IP moat” secures market exclusivity not just for the drug itself but for the means of its production, a strategy that has proven highly effective in the biopharmaceutical industry. It transforms the production platform into a strategic asset, enhancing the overall valuation of the development program.

### The economic calculus: balancing upfront CAPEX against long-term viability

Critically, the shift to a microbial platform fundamentally alters the economic structure of drug production. Traditional plant extraction follows a “Low CAPEX/High OPEX” model: initial development costs are minimal, but marginal costs scale linearly with agricultural volume, and downstream processing (DSP) remains a dominant cost driver, often accounting for 50-80% of the Cost of Goods Sold (COGS) due to the complexity of purifying low-titer compounds from complex plant matrices (Ghawte et al., 2025; Nandi et al., 2016).

In contrast, the decoupled microbial model represents a “High CAPEX/Low OPEX” strategy. It necessitates a significant upfront investment in strain engineering to manage metabolic burden and achieve commercially viable titers (>1 g/L) (Lynch, 2021). However, this upfront cost acts as a gatekeeper to long-term profitability. Once a high-performance strain is developed, production scales with the efficiency of industrial fermentation, where economies of scale can dramatically reduce unit costs (Lynd et al., 2022). For high-value pharmaceuticals, this scalability is essential. While the “fit-for-purpose” use of plant extraction may remain economically viable for low-value nutraceuticals where supply chains are robust, the high margins and stringent purity required for clinical therapeutics heavily favor the controllable, scalable economics of the microbial chassis (Juneja et al., 2019; Oraby et al., 2022).

### Case study: a tale of two development paths

Consider the decades-long struggle to produce paclitaxel, reliant on a slow-growing and finite natural source. The dependency on yew tree bark led to supply shortages, environmental concerns, and high costs, delaying its widespread availability to patients (Cragg, 1998; Yin et al., 2025). Now, imagine a new candidate with similar promise discovered tomorrow. Under the old model, it would face the same supply and consistency nightmare. Under this new paradigm, the validated pathway is immediately transferred to a scalable production host (Beekwilder et al., 2024; Zhang et al., 2025).

This isn’t just an improvement; it’s a fundamental acceleration, turning a 20-year development cycle into one that could take just a few years. By decoupling discovery from production, we bypass the botanical bottlenecks that have historically plagued natural product development (Chen et al., 2019; Van Dien, 2013). We move from a reliance on nature’s whims to a controlled, industrial process that can be scaled rapidly to meet global demand (Helf et al., 2024).

Therefore, the paradigm shift is complete. We move from fighting biological instability to leveraging it for discovery, and from there to imposing rigorous design for production. This creates a virtuous cycle: the predictability of the production platform de-risks commercial investment, which in turn funds the powerful discovery engine.

This new paradigm, integrating AI-driven discovery with predictable production, thus resolves the fundamental tension between biological complexity and industrial rigor. It offers a cohesive and transformative vision for the future of plant-inspired therapeutics, turning a century-old field of challenges into a frontier of unprecedented opportunity.

## Section H: synthesis and future perspectives—a new mandate for plant biotechnology

This review has articulated a fundamental shift, moving from a century-old paradigm to a new, technologically-enabled mandate for plant biotechnology. The journey began by confronting a fundamental truth: the biological instability of plant systems is not a flaw to be engineered away, but a fundamental property to be understood and leveraged. The inherent plasticity of plant metabolism, driven by complex environmental and genetic interactions, has historically been the stumbling block for consistent biomanufacturing in native hosts (Davies, 2010; Van Dien, 2013).

The logical arc of this paradigm shift traces a path from the “Sisyphean Endeavor” of the old control paradigm to the reframing of variability as a “Discovery Engine.” Rather than suppressing the chaotic response of plant cells to stress, the new model induces and amplifies it to access a broader chemical space (Kurita and Linington, 2015; Schenck and Last, 2020). This “productive chaos” is then decoded by an AI-powered toolkit, transforming unintelligible spectral data into a prioritized road map of bioactive molecules (Beniddir et al., 2021; Mullowney et al., 2023). Finally, the pragmatic blueprints for stable production (Section E) and predictive design (Section F) decouple discovery from manufacturing. This establishes an integrated, self-reinforcing pipeline where discovery informs production, and the predictability of the production platform de-risks the discovery process (Choi et al., 2019; Ke and Yoshikuni, 2020).

### The new interdisciplinary mandate

This new paradigm demands a new kind of scientist. The plant biotechnologist of the future must be a polyglot, fluent in the languages of molecular biology, analytical chemistry, data science, and synthetic biology (Mosoh and Vendrame, 2026). The convergence of these fields requires training paradigms that bridge the gap between wet-lab experimentation and computational modelling (Espinel-Ríos, 2025; Wu et al., 2016). Our institutions must foster this interdisciplinary convergence, creating collaborative structures where a metabolic engineer can seamlessly interface with an AI specialist to iterate on strain design (Chen J. et al., 2025). The “bio-digital” scientist will not merely observe biological systems but will actively interrogate and redesign them using a synthesis of biological intuition and computational precision (Volk et al., 2020).

### The grand challenge agenda

Our path forward is clear, but not without hurdles. Three distinct categories of challenges must be addressed to fully realize this vision:

- Biological Hurdles: We must conquer the stubborn challenge of functionally expressing complex plant enzymes, particularly membrane-bound cytochrome P450s, in industrial microbial hosts. While yeast offers a suitable membrane environment, optimizing the redox partners and protein folding for high-titer production remains a critical bottleneck (Cravens et al., 2019; Paddon et al., 2013).
- Data Hurdles: The power of AI is limited by the quality and quantity of data available for training. There is a critical need for larger, curated, open-access datasets—encompassing MS/MS spectra, genomic sequences, and bioactivity profiles—to fuel the next generation of predictive models (Jarmusch et al., 2021; Wang et al., 2023). Initiatives to standardize metadata and promote data sharing are essential to build the “public commons” required for robust machine learning applications (Rana et al., 2020).
- Integration Hurdles: Finally, we must engineer the integrated digital platforms that turn our pipeline from a series of discrete steps into a seamless, intelligent whole. Developing unified software frameworks that connect the AI-driven discovery engine directly with predictive design tools for strain engineering will create a truly continuous “design-build-test-learn” workflow, accelerating the translation of discoveries into products (HamediRad et al., 2019; Lawson, 2021; Lawson et al., 2019).

## Concluding vision

In conclusion, the convergence of biology, data science, and engineering marks a point of no return. The old strategy—a relentless, reactive battle against biological complexity—was a product of its time. Today, we have the tools to adopt a proactive, predictive posture. The future of natural product manufacturing is defined by precision: the precision of single-cell transcriptomics to find the needle in the haystack, the precision of AI to decode its structure, and the precision of synthetic biology to rebuild it in a microbial host. By trading the low upfront costs of extraction for the high-value investment of strain engineering, we break the linear cost barrier that has stifled the industry for decades. We stand at the threshold of a new era. By finally listening to and learning from biological complexity, rather than seeking to silence it, we can transition from struggling against living systems to partnering with them. This is the new mandate: to decode, harness, and redesign, thereby fully unlocking the profound chemical ingenuity of the plant kingdom for human health and beyond (**Box 6**).

Box 6 **Highlights, outstanding questions, and glossary**

Highlights

- The Scalability Trap: Undifferentiated plant cell cultures are intrinsically unsuited for industrial fermentation due to epigenetic drift, resulting in a "Low CAPEX / High OPEX" trap where production costs scale linearly with volume.
- Chaos as a Resource: Rather than suppressing stress, the new paradigm uses elicitation to generate a "productive chaos" of chemical diversity, transforming biological instability into a discovery engine.
- Single-Cell Resolution: Multiplexed single-cell transcriptomics and metabolomics decode metabolic heterogeneity, isolating rare "elite" producer cells to identify high-flux gene clusters hidden in bulk data.
- Architectural Engineering: Functional reconstitution in microbial hosts requires systems-level precision, including N-terminal engineering of P450s, optimization of P450:CPR stoichiometry, and global cofactor balancing.
- The Hairy Root Alternative: For pathways too complex for microbial reconstruction, differentiated hairy root cultures provide a genetically stable, tissue-specific "Plan B" chassis.
- Economic Transformation: The framework replaces the unsustainable extraction model with a "High CAPEX / Low OPEX" microbial manufacturing platform, ensuring the scalability essential for high-value therapeutics.

Glossary:

1. Somaclonal Variation: The genetic and epigenetic variation detected in plants regenerated from tissue culture, often leading to phenotypic instability and loss of biosynthetic capacity in undifferentiated cell lines.
2. Productive Chaos: A conceptual framework proposing that stress-induced metabolic instability in plant cells should be maximized to access a broader chemical space for discovery, rather than minimized for production consistency.
3. Molecular Networking: A computational strategy (e.g., GNPS) that organizes mass spectrometry data by grouping spectra based on chemical similarity, allowing for the visualization of structural families and the identification of unknown analogues.
4. Heterologous Expression: The expression of a gene or gene pathway in a host organism (the chassis) that does not naturally contain the gene, typically to achieve higher yields or simplified purification.
5. Metabolic Context Matching: The strategic selection of a microbial production host (chassis) based on its innate metabolic capabilities (e.g., high acetyl-CoA flux) to support the specific demands of a target biosynthetic pathway.
6. Design-Build-Test-Learn (DBTL) Cycle: An iterative engineering workflow used in synthetic biology where strains are designed *in silico*, constructed via genetic engineering, phenotyped, and the resulting data used to inform the next round of design.
7. Biofoundry: An integrated facility that combines robotics, automation, and data analytics to perform high-throughput strain construction and phenotyping, accelerating the DBTL cycle.
8. Flux Balance Analysis (FBA): A mathematical approach for analyzing the flow of metabolites through a metabolic network, used to predict the growth rate or production capabilities of an organism under steady-state conditions.
9. Generative AI: A class of artificial intelligence algorithms capable of generating new data instances (e.g., protein sequences, chemical structures) that resemble the training data, used for *de novo* enzyme and pathway design.
10. Techno-Economic Analysis (TEA): A methodology that evaluates the economic performance of a technological process, used here to contrast the linear cost scaling of plant extraction against the economies of scale offered by microbial fermentation.
11. CAPEX (Capital Expenditure) vs. OPEX (Operational Expenditure): Economic terms defining the cost structure of biomanufacturing. High CAPEX/Low OPEX refers to the microbial model (expensive upfront strain engineering, cheap scaling), whereas Low CAPEX/High OPEX refers to plant extraction (cheap setup, expensive scaling).
12. Transcriptional Bursting: The stochastic, pulsatile nature of gene expression that creates heterogeneity within a cell population; leveraged here as a mechanism to generate “elite” high-producing cells under stress.
13. Single-Cell Multi-Omics: The simultaneous profiling of multiple biological layers (e.g., transcriptomics and metabolomics) from the same single cell, allowing direct correlation between gene expression and metabolite accumulation.
14. Hairy Root Culture: A differentiated plant tissue culture induced by infection with Agrobacterium rhizogenes. Unlike undifferentiated cell suspensions, hairy roots maintain long-term genetic and biosynthetic stability, making them an ideal “Plan B” chassis for root-specific metabolites.
15. Cofactor Balancing: The engineering of metabolic flux to ensure an adequate supply and equilibrium of auxiliary molecules (e.g., ATP, NADPH, Acetyl-CoA) required for the catalytic activity of biosynthetic enzymes, particularly within confined organelles.
16. P450-CPR Stoichiometry: The precise ratio between a Cytochrome P450 enzyme and its redox partner, Cytochrome P450 Reductase (CPR). Optimization of this ratio is critical to prevent electron uncoupling and the generation of toxic Reactive Oxygen Species (ROS).

## Data Availability

The original contributions presented in the study are included in the article/supplementary material. Further inquiries can be directed to the corresponding author/s.
